# Characterization of preferential flow in soils near Zarqa river (Jordan) using in situ tension infiltrometer measurements

**DOI:** 10.7717/peerj.8057

**Published:** 2019-11-15

**Authors:** Michel Rahbeh

**Affiliations:** University of Jordan, School of Agriculture, Department of Land, Water and Environment, Amman, Jordan

**Keywords:** Macroporosity, Soil organic carbon, Macropore flux, Macroscopic capillary length

## Abstract

**Background:**

The Zarqa River (ZR) is located in the northern part of Jordan and supplies King Talal Dam (KTD). The streamflow that discharges into KTD is composed of treated wastewater from the Khirbat Es-Samra water treatment plant (KTP) and runoff generated during the winter season. Thus, during the summer, the streamflow of the ZR is dominated by effluent from the KTP. Due to the severe scarcity of water in Jordan, a portion of the streamflow is utilized for irrigated agriculture in the ZR valley, located between the KTP and KTD. The groundwater in the vicinity of the ZR is vulnerable to contamination—a risk that may be exacerbated by the potential occurrence of preferential flow (PF). Therefore, the PF in the soils near the ZR should be carefully considered.

**Methods:**

The macropore flux fraction (*Q*_macro_) and macroscopic capillary length (*λ*_*c*_) were determined from *in situ* measurements using a tension infiltrometer equipped with an infiltration disc with a diameter of 20 cm. The macropore was defined as the pore size that drains at a tension of less than —-3— cm. The *λ*_*c*_ less than 80 mm was considered to be an indication of PF. The measurements were taken at 69 sites along the ZR between the KTP and KTD. At each measurement site, the soil organic matter content (OM) and soil texture were determined using a composite soil sample obtained by excavating the soil beneath the infiltration disc to a depth of 10 cm.

**Results:**

The data was split into two groups: the matrix flow group (MF), which includes data associated with *λ*_*c*_ > 80 mm, and the PF group, which includes data associated with *λ*_*c*_ < 80 mm. The *Q*_macro_ values of 0.67 and 0.57, respectively, for PF and MF were significantly different at *p* < 0.01 (*t*-test). The flow rates at h=0 were generally well associated with *λ*_*c*_, as attested to by a significant difference between the averages of PF (57.8 mm/hr) and MF (21.0 mm/hr) at *p* < 0.01 (*t*-test). The OM was positively associated with PF. This was statistically confirmed by a *t*-test at *p* < 0.01. The average sand and clay contents of PF and MF were not statistically different. Analysis of the ratio of Soil Organic Carbon (SOC) to clay showed that the average SOC/clay of the PF (14%) was larger than that of the MF (13.3%). After the exclusion of soils with clay content less than 8%, the differences between the SOC/clay averages of PF (9.8%) and MF (7.5%) were significant at *p* < 0.05, as shown by a WM-test.

**Conclusion:**

The OM was positively associated with PF. Soil texture—and clay content in particular—influenced the *λ*_*c*_values. However, the association of clay content with PF was not statistically significant. Consideration of the SOC/clay ratio showed that the tendency toward PF increases as the complexation of the clay content increases. This was most obvious in soils with a clay content of greater than 8% and SOC/clay of approximately 10%. The OM either influences or is inter-correlated with the processes responsible for the formation of macropores.

## Introduction

**Figure 1:**
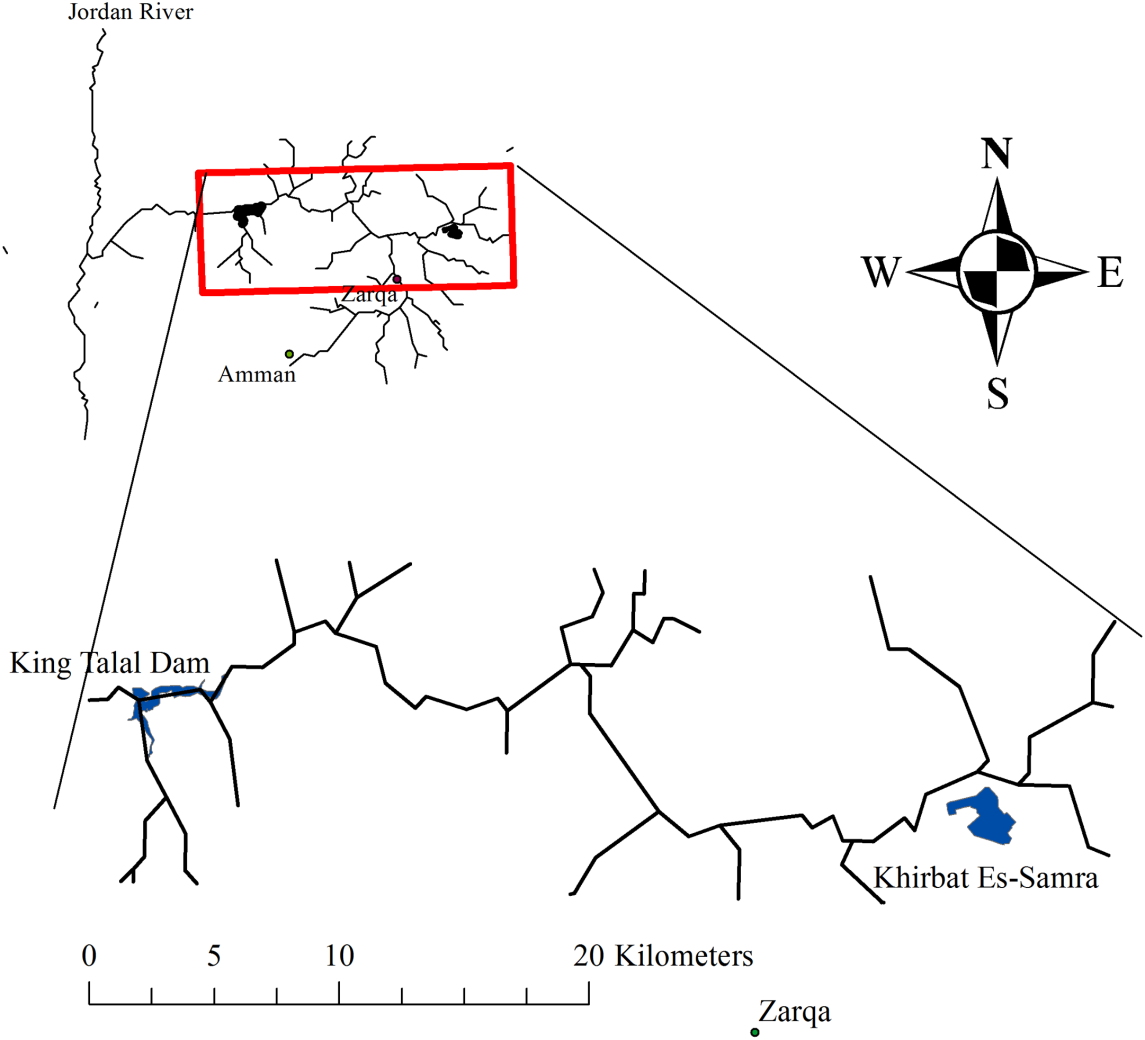
The location of the study area along the Zarqa River in Jordan.

The Zarqa River (ZR) is one of the major tributaries of the Jordan River, located in the northern part of Jordan. It is home to 50% of the Jordanian population. The streamflow of the ZR is composed of treated wastewater from the Khirbat Es-Samra water treatment plant (KTP) and natural runoff generated mainly from the northwestern part of the watershed. However, during the summer season, the streamflow of the ZR is dominated by effluent from the KTP and discharges into King Talal Dam (KTD) (Fig. 1). A portion of the streamflow is utilized upstream to irrigate the agricultural land located in the ZR valley. The utilization of treated wastewater for irrigation is expected to continue due to the well-documented scarcity of water in Jordan. The KTP wastewater treatment plant is the largest water treatment facility in Jordan and is located on a rechargeable groundwater zone (Al-Mahamid, 2005; Al-Kharabsheh, 1999). Bajjali, Al-Hadidi & Ismail (2017) reported rising groundwater levels in wells located near the KTP. Furthermore, Al-Kuisi et al. (2014) indicated that the groundwater between the KTP and KTD along the ZR is vulnerable to contamination, as evidenced by elevated nitrate concentrations. This raises the concern that solutes are leaching faster than anticipated through preferential pathways.

Hendrickx & Flury (2001) defined preferential flow as non-uniform water flow that causes water and solutes to bypass a fraction of the soil matrix. They also described three classes of preferential flow: (1) macropore flow, (2) unstable flow, and (3) funnel flow.

Unstable flow, also known as fingering, originates from a regular wetting front that breaks into fingers due to air entrapment, water-repellent soils, ponding, or continuous infiltration. Kapetas, Dror & Berkowitz (2014) strongly suggested that preferential flow can develop in uniform sandy soils as a result of successive wetting and drying cycles. Sheng et al. (2012) and Sheng et al. (2014) confirmed that the pathways originating from fingering are viable routes for preferential solute transport in silty clay and loamy soils. Hardie et al. (2011) reported finger flow, induced by hydrophobicity, that bypassed the topsoil horizon. The funnel flow also originates from a regular wetting front that develops into vertical pathways (funnels) due to the presence of coarse material within the fine-textured soil. Fox et al. (2011) observed that a preferential flow pathway composed of gravel and coarse material embedded in a flood plain facilitated the movement of water and solutes to the stream.

Preferential macropore flow occurs in many soils through macropores formed by roots and earthworm activity (henceforth, bio-pores), or through cracks and fissures resulting from shrinking clay (Beven & Germann, 1982). Bio-pores produced by the burrowing activity of ants and crickets facilitate the movement of water to deep soil layers (Li et al., 2018; Li et al., 2019). Macropores also enhance the mobility of solutes and nano-particles in sand and quartz (Fang et al., 2016; Benettin et al., 2019).

Zehe & Flühler (2001) suggested two main conditions for the occurrence of preferential macropore flow. The first condition is a sufficient number of interconnected macropores and the second condition is high initial soil water content. The formation of macropores is dependent on clay content (Iversen et al., 2012; Ghafoor et al., 2013) contingent on a threshold of 8–10% clay (Koestel, Moeys & Jarvis, 2012; Jarvis, Koestel & Larsbo, 2016). Dexter et al. (2008) suggested that the ratio between Soil Organic Carbon (SOC) and clay contents affects the soil’s hydraulic properties more than the total clay content or total organic matter content (OM) does. They showed that 1 g of SOC is needed to fully complex 10 g of clay. For pasture and organic-rich soils, the reciprocal of bulk density was correlated with clay content, while for arable or soil of low OM, the reciprocal of bulk density was correlated with OM. The SOC/clay ratio of 10% for full complexation of clay was confirmed by Larsbo et al. (2016). They observed that, for soil with high clay and OM, preferential transport was not affected when the SOC/clay ratio was greater than 10%.

Dye tracer experiments (Wang & Zhang, 2011) showed that macropore flow patterns develop during the infiltration process and the subsequent increase in the soil water content. However, the tracer experiments conducted by Flury et al. (1994) and Villholth, Jensen & Fredericia (1998) showed that the initial water content had a minimal effect on the preferential macropore flow. Nimmo (2012), citing field observation and experimental evidence, pointed out that flow through bio-pores can be initiated under unsaturated conditions. Field investigation of subsurface water movement conducted on a hillslope scale revealed that percolating water bypassed the soil profile through macropores; consequently, the lateral flow may not develop within the soil layers but, rather, starts at the interface between the soil and the bedrock (Smettem et al., 1991; Buttle & McDonald, 2002).

Wuest (2009) observed preferential flow pathways in tilled and untilled soils; however, preferential flow zones were of greater proportion in untilled soils than in tilled soils. Malone et al. (2003) showed that tillage did not significantly change the number of macropores; rather, it altered the hydraulic conductivity and porosity, resulting in earlier breakthrough curves and higher concentrations in untilled soils compared to tilled soils. Ghafoor et al. (2013) also showed that preferential flow and transport occur in tilled soils. Williams et al. (2016) observed that the effect of disk tillage on macropore flow diminished in less than three weeks after the tillage operation. This was attributed to the formation, in the tilled soils, of cracks caused by raindrops and wetting and drying cycles. Worms can also re-establish bio-pores in a plowed soil layer (Andreini & Steenhuis, 1990).

Several methods—including field investigations using dye tracers, transport experiments on undisturbed soil columns, and X-ray CT scans of intact soil core—have been used in the characterization of preferential flow (Allaire, Roulier & Cessna, 2009). These methods are vital to the study of preferential flow and transport; however, they require a considerable amount of time and effort, which may inhibit their use in spatial scale investigations of macropore flow. Alternatively, in situ measurements using a tension infiltrometer may be a more appropriate choice for large-scale investigations because they allow for a relatively quick and reliable determination of soil macropore flux (Watson & Luxmoore, 1986; Holden, Burt & Cox, 2001). In fact, in situ measurements using a tension infiltrometer were systematically used in the evaluation of preferential flow and transport characteristics (Buttle & McDonald, 2002; Tallon, Si B. C. Korber & Guo, 2007; Kodesova et al., 2010).

The full extent and impact of preferential flow on the ZR is unknown. Therefore, the environmental risk of preferential flow in the farm area adjacent to the ZR should be carefully considered, especially given that the groundwater in the vicinity of the ZR is vulnerable to contamination. This paper aims to evaluate the potential of preferential flow along the ZR by (1) testing for the presence or absence of preferential flow and (2) evaluating the effect of OM and soil texture on preferential flow.

## Material and Methods

### Study site

The study area between the KTP and KTD (Fig. 1) contains forage and vegetables, as well as olive and citrus orchards. The farmland is 300–500 m above sea level. The relatively warm weather, characterized by a maximum temperature range of 25 to 46 °C and an average temperature range of 19 °C, enables farmers to undertake 2 or 3 cropping cycles per year. Farmers follow conventional tillage practices, using a moldboard/chisel plow to dig to a depth of 10–15 cm and, in some fields, harrowing the topsoil. Farmers usually till their fields before each cropping cycle, typically during the fall (September/October) or early spring (March/April).

The KTP is used to irrigate the agriculture lands adjacent to the main reach of the ZR. The average annual rainfall depth of less than 150 mm constitutes a small fraction of total crop water requirements. Hence, agricultural practices depend mainly on irrigation water supplied directly from the river or local wells.

**Figure 2:**
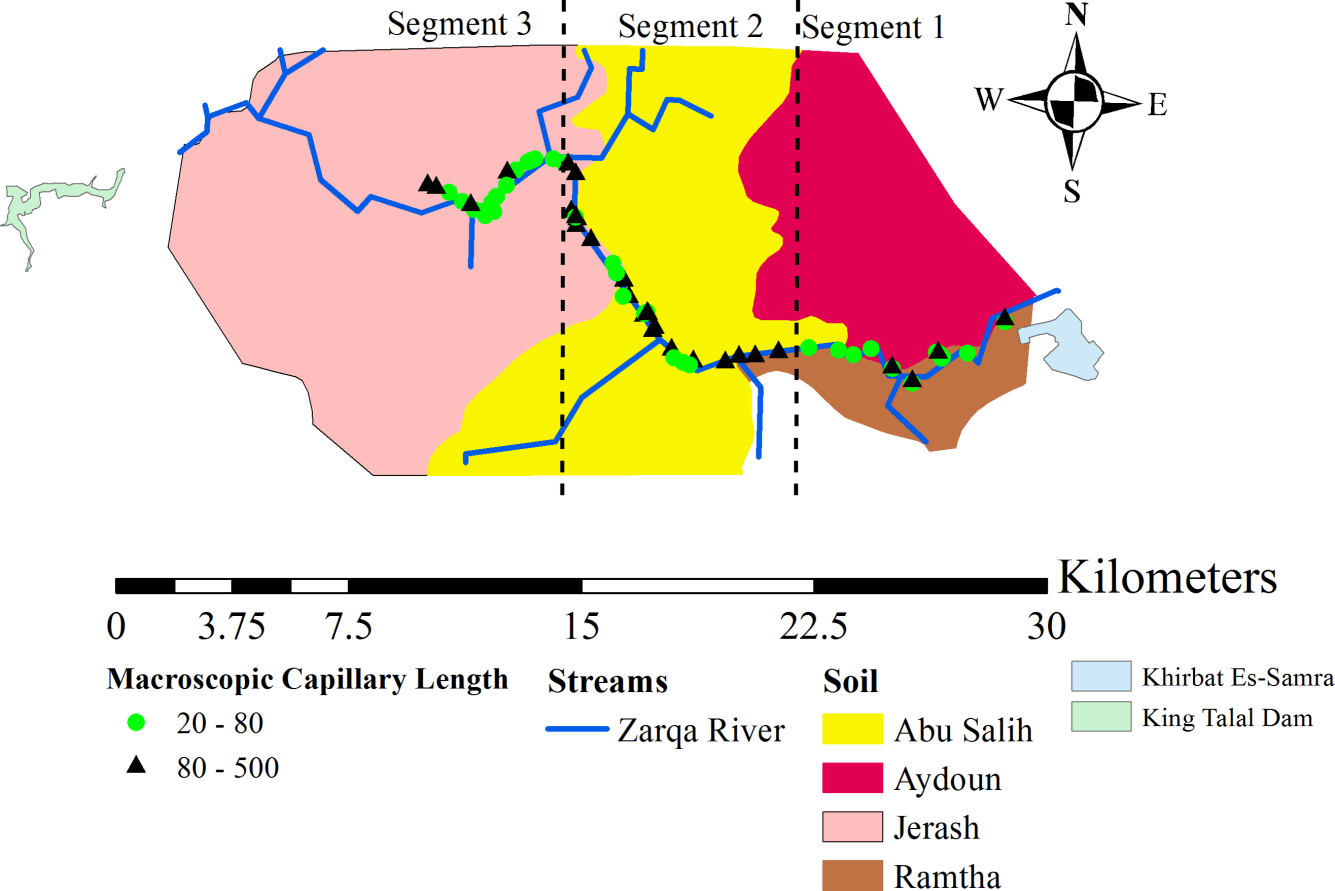
The locations of the measurement sites and the soil units encountered around the Zarqa River. The map is subdivided into three sections to reflect the differences between the *λ*_*c*_ measurements along the river.

Four types of soils exist within the study area (Fig. 2). These are Jerash (JER), Abu Salih (ALI), Aydoun (AYD), and Ramtha (THA). These soils belong to the Lithic and Typic Xerorthents, and Xerochreptic Camborthids subgroups (Table 1). The JER, ALI, and AYD overlay geologic formations that consist mainly of limestone and chalky limestone. Meanwhile, the THA sits on a basalt formation (Al-Mahamid, 2005).

**Table 1:** Description of the soil units encountered in the study area (Ministry of Agriculture of Jordan, 1994).

**Soil Unit**	**Name**	**Sub group**	**Texture**	*T*_mean_(°C)	**Annual rainfall** (mm)	**Parent material**	**Geology**
JER	Jerash	Lithic Xerorthents	Clay loam	17.0	300–350	Colluvium	Sedimentary: Limestone
ALI	Abu Salih	Typic Xerorthents	Sandy Clay Loam	18.8	200–250	Alluvium	Unconsolidated alluvium: Limestone
AYD	Aydoun	Typic Xerochrepts	Silty clay loam	17.8	150–200	Alluvium	Unconsolidated alluvium: Limestone
THA	Ramtha	Xerochreptic Camborthids	Silty loam	17.4	15–200	Alluvium	Basalt

### Tension infiltrometer measurements

A standard tension infiltrometer equipped with an infiltration disc that was 20 cm in diameter, a pressure transducer, and a data logger (supplied by ICT International) similar to the device described by Ankeny, Kaspar & Horton (1988) were used. A total of 69 measurements were taken from May through September of 2016. The measurements were made during the summer growing season, at least one month after seedbed preparation and plowing activity. At each location, the upper layer of loose soil and plant residue was removed. The soil surface was then leveled and a thin layer of silica sand was placed under the infiltrometer disc to maintain hydraulic contact between the infiltration disc and the soil surface. The measurements were taken sequentially at three tensions of −6, −3, and −0 cm. The −0 indicates that the tension infiltrometer was adjusted to a slight tension to prevent the free flow of water from the tension disc. The water intake was recorded at intervals of one minute. The measurements were allowed to proceed until a steady infiltration rate had been attained, which took about 20 to 60 min for each tension. Thus, the tension infiltrometer measurements can be interpreted according to the following steady-state flow equation (Wooding, 1968):

(1) \begin{eqnarray*}Q \left( {h}_{i} \right) =\pi {r}^{2}{K}_{s}\exp \nolimits (\alpha {h}_{i}) \left( 1+ \frac{4}{\pi r\alpha } \right) \end{eqnarray*}

where *Q*(*h*_*i*_) is the flow rate at tension *h*_*i*_(*L*^3^∕*T*), *K*_*s*_ is the saturated hydraulic conductivity (*L*∕*T*), *α* is the sorptive number (1∕*L*), and *r* is the radius of the tension infiltrometer disc (L).

Considering two data points at *h*_1_ =  − 6 cm and *h*_2_ =  − 3 cm, and taking *K*_*s*_ as a constant, then dividing the *Q*(*h*_2_) equation by *Q*(*h*_1_) and solving for *α*:

(2) \begin{eqnarray*}\propto = \frac{\ln \nolimits \left[ Q \left( {h}_{2} \right) /Q({h}_{1}) \right] }{{h}_{2}-{h}_{1}} .\end{eqnarray*}

The −6 to −3 tension range was used for the calculation of *α* instead of the −3 to −0 tension range because the infiltration rate and *K*_*s*_ increase rapidly as the tension approaches zero. This may lead to the overestimation of *α* values if they were derived from a tension range close to saturation (Clothier & Smettem, 1990; Jarvis & Messing, 1995).

The first measurement location was near the KTP, while additional sites were selected along the westward course of the river (Fig. 2). However, the locations were not necessarily visited sequentially; some back-and-forth occurred due to issues with accessibility and with navigating the terrain of the ZR. The geographic coordinates were recorded using a Garmin Etrex 30 GPS device.

### Susceptibility to preferential macropore flow

The physical characterization of preferential flow is generally related to macroporosity, defined as the ratio of the total volume of macropores to the total volume of soil (e.g., Beven & Germann, 1982; Luo, Lin & Schmidt, 2010; Mossadeghi-Björklund et al., 2016). Indeed, this is a beneficial parameter. However, the definition of macropores is rather arbitrary. For example, Beven & Germann (1982) reported that values between 30 to 10,000 µm represented the effective macropore diameter, or the equivalent of −102 to 1.02 cm tension. Jarvis (2007) indicated that pores with diameters larger than 300 µm can conduct rapid non-equilibrium flow. Watson & Luxmoore (1986) defined macropores as pores that conduct flux at tension less than | − 3| cm, or equivalent to a pore radius greater than 500 µm. They found that three-quarters of the flux in tension infiltrometers occurs between 0 and −3 cm tension. Another useful parameter is macroscopic capillary length (*λ*_c_) which is equivalent to the wetting front suction in the Green and Ampt equation. It is also equivalent to the inverse of *α* determined by Eqn. (2) (Nachabe, 1996).

White & Sully (1987) compared the *λ*_*c*_ for disturbed and undisturbed soils. They found that *λ*_*c*_ values increased drastically in disturbed soils. Furthermore, their results showed that *λ*_*c*_ values of less than 80 mm were considered indicators of preferential flow. For the purpose of this study, two definitions were adopted. First, a macropore is a pore that drains at less than —−3— cm tension. Second, preferential flow is indicated by *λ*_*c*_ values of less than 80 mm.

Accordingly, the macropore flux fraction (*Q*_macro_), defined as the fraction of the flux occurring between *h* =  − 3 cm and *h* = 0 cm, was determined by normalizing the difference between *I*(*h* =  − 0cm) and *I*(*h* =  − 3cm) with respect to *I*(*h* =  − 0cm), where I is the infiltration rate (mm/hr) (Watson & Luxmoore, 1986).

### Soil sampling

After each measurement, a composite soil sample was collected from a hole excavated beneath the tension disk to a depth of 10 cm. The collected samples were analyzed for the OM and soil texture distribution. The OM was determined using the Walkley–Black (WB) method (Walkley & Black, 1934). The WB method measures the SOC. The OM is calculated by using a standard ratio of 1.72 between the OM and the SOC (i.e., OM = 1.72*SOC). The soil textural classes were determined using the hydrometer method (Bashour & Sayegh, 2007).

## Results

### Exploratory data analysis

**Figure 3:**
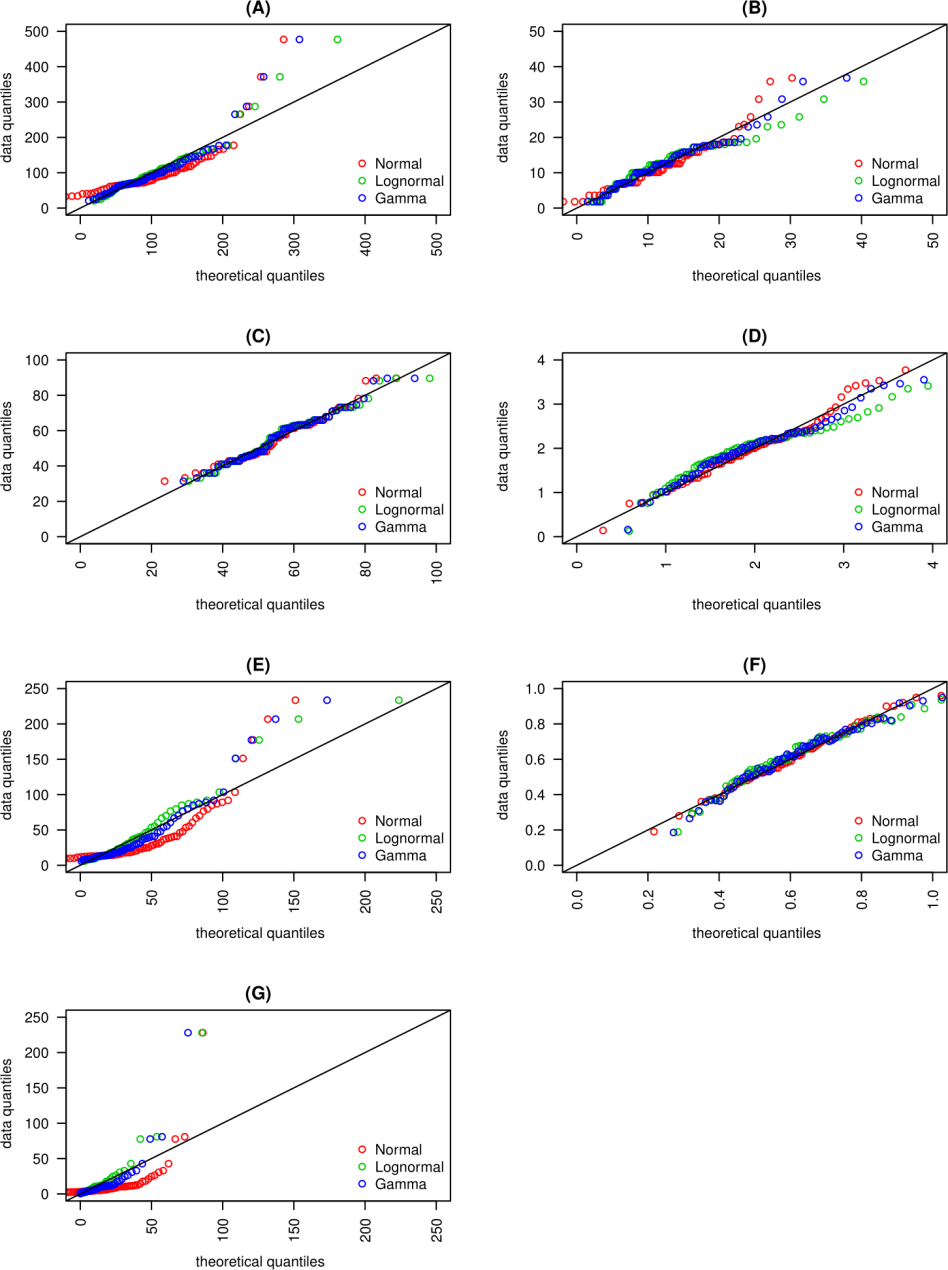
Quantile- Quantile plots for (A) macroscopic capillary length, (B) clay content, (C) sand content, (D) organic matter content, (E) infiltration rate, (F) macropore fraction and, (G) hydraulic conductivity are presented. Quantile- Quantile plots are fitted to normal, lognormal and gamma distributions.

**Figure 4:**
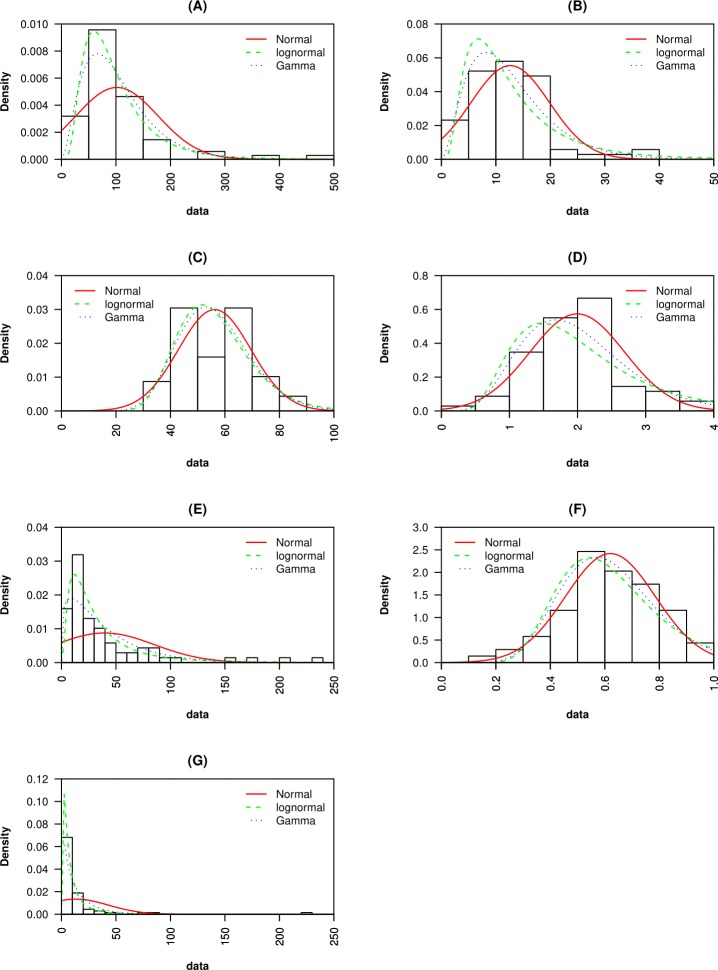
Probability density functions for (A) macroscopic capillary length, (B) clay content, (C) sand content, (D) organic matter content, (E) infiltration rate, (F) macropore fraction and, (G) hydraulic conductivity are presented. Probability density functions are fitted to normal, lognormal and gamma distributions.

**Figure 5:**
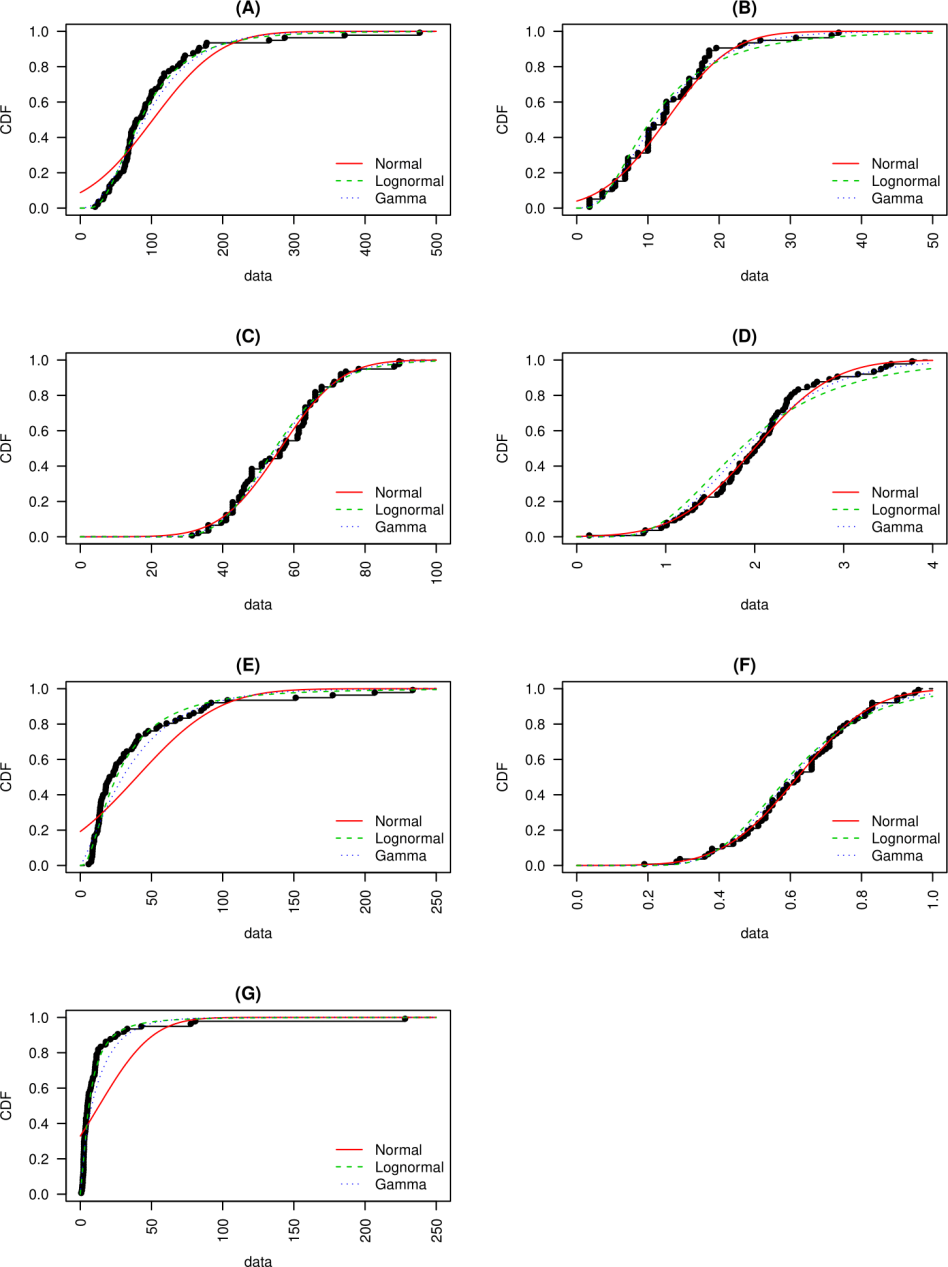
Cumulative distribution functions for (A) macroscopic capillary length, (B) clay content, (C) sand content, (D) organic matter content, (E) infiltration rate, (F) macropore fraction and, (G) hydraulic conductivity are presented. Cumulative distribution functions are fitted to normal, lognormal and gamma distributions.

All data sets were tested for normality using the Shapiro–Wilk (SK) and Anderson-Darling (AD) normality tests (Table 2). Furthermore, each data set was compared to quantile–quantile plots (QQ-plots), the probability density function (PDF), and the cumulative distribution function (CDF) of the normal, lognormal, and gamma distributions (Figs. 3–5). Statistical analysis was performed by R language version 3.4.3.

**Table 2:** The *p*-values of the SW and AD normality test for *λ*_*c*_, OM, *I*(*h* =  − 0cm), *Q*_macro_, *K*_*s*_ and, clay and sand contents. The null hypothesis of the normal distribution is rejected at *p* < 0.05.

	Non transformed data		lognormally transformed data	
**Data**	**SW**	**AD**	**SW**	**AD**
*λ*_*c*_	0.00	0.00	0.37	0.26
OM	0.39	0.22	0.00	0.00
OM (*λ*_*c*_ < 80 mm)	0.30	0.34	0.58	0.80
OM (*λ*_*c*_ ≥ 80 mm)	0.13	0.42	0.16	0.07
Clay content	0.00	0.01	0.00	0.00
Clay (*λ*_*c*_ < 80 mm)	0.01	0.09	0.07	0.06
Clay (*λ*_*c*_ ≥ 80 mm)	0.02	0.04	0.02	0.02
Sand content	0.06	0.07	0.22	0.07
Sand (*λ*_*c*_ < 80 mm )	0.14	0.13	0.22	0.07
Sand (*λ*_*c*_ ≥ 80 mm)	0.32	0.31	0.51	0.53
*K*_*s*_ (mm/hr)	0.00	0.00	0.02	0.03
*K*_*s*_ (*λ*_*c*_ < 80 mm )	0.00	0.00	0.05	0.05
*K*_*s*_ (*λ*_*c*_ ≥ 80 mm)	0.00	0.00	0.75	0.47
*I*(*h* = 0cm) (mm/hr)	0.00	0.00	0.01	0.01
*I*(*h* = 0cm)(*λ*_*c*_ < 80mm)	0.00	0.00	0.83	0.90
*I*(*h* = 0cm)(*λ*_*c*_ ≥ 80mm)	0.00	0.00	0.00	0.00
*Q*_**macro**_	0.93	0.98	0.00	0.02
*Q*_**macro**_(*λ*_*c*_ < 80mm)	0.36	0.51	0.04	0.00
*Q*_**macro**_(*λ*_*c*_ ≥ 80mm)	0.78	0.46	0.00	0.27

The SK and AD tests returned *p*-values greater than 0.05 for OM, *Q*_macro_, and sand content. For these data sets, the null hypothesis of normality cannot be rejected at *p* < 0.05 (Table 2). The QQ-plot (Fig. 3) showed that OM data fits the normal distribution better than it does the lognormal or gamma distributions. This was evidenced by the oscillation of QQ points of the lognormal and gamma distributions around the 1:1 reference line (defined as a straight line of intercept = 0 and slope = 1), while the QQ points of the normal distribution fell adjacent to the reference line except for a few extreme values. The CDF (Fig. 5) and QQ-plots (Fig. 3) of *Q*_macro_ and sand content did not include clear indications that distinguished the normal distribution from the lognormal and gamma distributions. However, the PDF (Fig. 4) of the normal distribution fits the peaks, the right and left tails of OM, *Q*_macro_, and the sand content histograms better than the lognormal and gamma distributions.

The normality of the clay data was not confirmed by either SK or AD tests. Further examination of the clay QQ-plots (Fig. 3) showed that normal distribution exhibited the least oscillation around the reference line. Similar to the OM, *O*_pore_, and sand content data, the PDF of the normal distribution reasonably fits the histogram of the clay content data.

The lognormal distribution PDF (Fig. 4) and CDF (Fig. 5) produced the best fit of the *λ*_*c*_ data. In fact, SK and AD (Table 2) confirmed the normal distribution of the log-transformed *λ*_*c*_ data. It was anticipated that the saturated hydraulic conductivity (*K*_*s*_) and *I*(*h* =  − 0cm) are also lognormally distributed. However, normality for the log-transformation of both data sets was not confirmed by either SK or AS, possibly due to the tailing caused by extreme values. Nevertheless, the CDF plots (Fig. 5) of the *K*_*s*_ and *I*(*h* =  − 0cm) data suggest a reasonable fit of the lognormal or gamma distributions. Further inspection of the PDF plot (Fig. 4) shows that lognormal distribution properly represents the positively skewed *K*_*s*_ and *I*(*h* =  − 0cm) histograms.

Thus, the *t*-test may be conducted for the OM, sand and clay, *Q*_macro_, and the log-transformed data of *K*_*s*_ and *I*(*h* =  − 0cm). However, non-parametric data analysis (Mann–Whitney U test, hereafter MW-test) was also applied to all data sets because the SK and AD test did not confirm the normality of clay content and the log-transformed hydraulic conductivity and *I*(*h* =  − 0cm).

### In situ tension infiltrometer measurements

The exploratory analysis showed an average *λ*_*c*_ value of 102 mm and a geometric mean of 84 mm, which conforms with the average values that White & Sully (1987) suggested. The *λ*_*c*_ also showed a standard deviation of 75 mm and a positive skewness of 2.8 (Table 3).

**Table 3:** The statistical parameters and the tests of significance (*t*-test and MW-test) for *λ*_*c*_, OM, *I*(*h* =  − 0cm), *Q*_macro_, *K*_*s*_ and, clay and sand contents are presented. Significant differences are indicated at *p* < 0.05.

					Non transformed data		Lognormally transformed data	
**Data**	**A.M.**	**SD**	**Skewness**	**G.M.**	***t*-test**	**MW-test**	***t*-test**	**MW-test**
*λ*_*c*_ (mm)	101.9	75.7	2.7	84.3				
*λ*_*c*_ < 80 mm	57.0	17.0	−0.7	53.9				
*λ*_*c*_ ≥ 80 mm	148.1	84.7	2.6	133.1				
OM	2.0	0.7	0.2	1.8				
OM (*λ*_*c*_ < 80 mm)	2.2	0.7	0.4	2.2	*t*(67) = 2.91, *p* = 0.005	*p* = 0.009	*t*(61) = − 2.88, *p* = 0.005	*p* = 0.010
OM (*λ*_*c*_ ≥ 80 mm)	1.8	0.6	0.6	1.7				
Clay	12.6	7.3	1.1	10.5				
Clay (*λ*_*c*_ < 80 mm)	12.6	6.7	1.2	10.9	*t*(65) = − 0.03, *p* = 0.974	*p* = 0.732	*t*(63) = − 0.43, *p* = 0.666	*p* = 0.732
Clay (*λ*_*c*_ ≥ 80 mm)	12.7	7.9	1.0	10.1				
Sand	56.3	13.4	0.4	54.7				
Sand (*λ*_*c*_ < 80 mm)	56.7	13.9	0.4	55.1	*t*(67) = 0.30, *p* = 0.767	*p* = 0.890	*t*(67) = − 0.24, *p* = 0.812	*p* = 0.890
Sand (*λ*_*c*_ ≥ 80 mm)	55.8	13.1	0.4	54.3				
*K*_*s*_ (mm/hr)	13.2	30.0	5.6	6.0				
*K*_*s*_ (*λ*_*c*_ < 80 mm)	23.0	40.0	4.4	12.8	*t*(34) = 2.94, *p* = 0.006	*p* = 0.000	*t*(54) = 8.45, *p* = 0.00	*p* = 0.000
*K*_*s*_ (*λ*_*c*_ ≥ 80 mm)	3.1	1.8	2.1	2.7				
I (mm/hr)	39.7	45.9	2.4	25.4				
I (*λ*_*c*_ < 80 mm)	57.8	53.0	1.9	42.1	*t*(51) = 3.65, *p* = 0.001	*p* = 0.000	*t*(66) = 5.77, *p* = 0.000	*p* = 0.000
*I*(*λ*_*c*_ ≥ 80mm)	21.0	27.2	3.6	15.1				
*Q*_macro_	0.6	0.2	0.1	0.6				
*Q*_macro_(*λ*_*c*_ < 80mm)	0.7	0.2	0.5	0.6	*t*(67) = 2.45, *p* = 0.017	*p* = 0.009	*t*(66) = − 2.26, *p* = 0.027	*p* = 0.009
*Q*_**macro**_(*λ*_*c*_ ≥ 80mm)	0.6	0.2	0.2	0.5				

Three segments can be distinguished based on the number of *λ*_*c*_ observations below and above the critical value (Fig. 2). The first segment starts near the vicinity of the KTP and then extends approximately 7 kilometers (km) to the west. The second segment extends 3 km farther downstream, then meanders northwest to an approximate distance of 7 km. The third segment is considered the point where the river starts moving in a westward direction. Figure 2 suggests that the first and third sections are dominated by *λ*_*c*_ observations below the critical value.

It is also beneficial to consider observations at the locations where the above critical values of *λ*_*c*_ were observed separately from the rest of the observations. Therefore, the determination of the average values of the observed parameters proceeded with a ranking of the data with respect to *λ*_*c*_ value, then the establishment of two groups by splitting the record at the *λ*_*c*_ critical value (Table 4). This was done for each segment as well as for the entire study area. Thus, the data included in the first group (a total of 35 samples) is associated with the preferential flow, henceforth referred to as PF, as indicated by *λ*_*c*_ of less than 80 mm, specifically, *λ*_*c*_ geometric means of 43.9, 64.6, 54.2, and 53.9 mm for segments 1, 2, 3, and the whole area, respectively (Tables 3 and 4). By contrast, the *λ*_*c*_ values for the second group (a total of 34 samples), or the matrix flow group (MF), are 2 to 3 times greater than that of PF. Furthermore, an unequal variances student’s *t*-test and an MW-test were performed on the groups representing the entire study area (Table 3). Both tests were not performed on the *λ*_*c*_ values because, by definition, the average *λ*_*c*_ of PF (data associated with *λ*_*c*_ < 80 mm) should be less than the average of MF (data associated with *λ*_*c*_ ≥ 80 mm). The geometric means of *λ*_*c*_ representing the whole study area were 53.9 and 133.71 mm for PF and MF, respectively.

**Table 4:** The arithmetic (A.M.) and geometric (G.M.) means for *λ*_*c*_, OM, *I*(*h* =  − 0cm), *Q*_macro_, *K*_*s*_ and, clay and sand contents located within segments 1, 2 and 3.

	Segment 1		Segment 2		Segment 3	
	A.M.	G.M.	A.M.	G.M.	A.M.	G.M.
*λ*_*c*_ (mm) (All)	81.5	63.0	111.9	101.6	100.3	77.3
*λ*_*c*_ (mm) (*λ*_*c*_ < 80 mm)	47.2	43.9	65.6	64.6	57.2	54.2
*λ*_*c*_ (mm) (*λ*_*c*_ ≥ 80 mm)	167.2	155.7	136.2	128.8	167.3	136.4
OM (All)	2.4	2.2	2.0	1.9	1.7	1.5
OM (*λ*_*c*_ < 80 mm)	2.9	2.8	2.3	2.3	1.7	1.7
OM (*λ*_*c*_ ≥ 80 mm)	1.4	1.3	1.9	1.8	1.6	1.3
I (*h* = 0 cm) (mm/hr) (All)	85.6	51.7	26.2	19.0	30.5	24.7
I (*h* = 0 cm) (mm/hr) (*λ*_*c*_ < 80 mm)	114.9	92.6	27.0	22.9	41.3	38.7
I (*h* = 0 cm) (mm/hr) (*λ*_*c*_ ≥ 80 mm)	12.1	12.0	25.8	17.3	13.5	12.3
I (*h* = − 3 cm) (mm/hr) (All)	18.8	12.7	6.7	6.0	12.0	10.6
I (*h* = − 3 cm) (mm/hr) (*λ*_*c*_ < 80 mm)	23.8	16.9	8.2	7.6	14.9	13.8
I (*h* = − 3 cm) (mm/hr) (*λ*_*c*_ ≥ 80 mm)	6.4	6.3	5.8	5.3	7.6	7.0
I (*h* = − 6 cm) (mm/hr) (All)	8.5	7.1	4.8	4.4	7.4	6.7
I (*h* = − 6 cm) (mm/hr) (*λ*_*c*_ < 80 mm)	9.8	8.0	5.2	4.7	8.3	7.6
I (*h* = − 6 cm) (mm/hr) (*λ*_*c*_ ≥ 80 mm)	5.2	5.0	4.6	4.2	6.0	5.4
*Q*_macro_ (All)	0.68	0.65	0.66	0.64	0.54	0.52
*Q*_macro_ (*λ*_*c*_ < 80 mm)	0.75	0.73	0.65	0.63	0.62	0.60
*Q*_macro_ (*λ*_*c*_ ≥ 80 mm)	0.47	0.47	0.66	0.64	0.42	0.40
Clay content (All)	13.5	10.8	14.9	13.8	8.9	7.0
Clay content (*λ*_*c*_ < 80 mm)	15.8	12.4	11.8	11.4	11.0	9.5
Clay content (*λ*_*c*_ ≥ 80 mm)	8.0	7.6	16.6	15.3	5.6	4.4
Sand content (All)	53.0	51.5	53.5	52.5	62.1	60.1
Sand content (*λ*_*c*_ < 80 mm)	50.4	48.7	59.8	59.2	58.9	56.8
Sand content (*λ*_*c*_ ≥ 80 mm)	59.4	59.1	50.2	49.2	67.2	65.7
*K*_*s*_ (mm/hr) (All)	35.0	12.0	4.4	3.5	12.1	8.0
*K*_*s*_ (*λ*_*c*_ < 80 mm)	47.9	22.4	7.3	6.6	17.5	14.5
*K*_*s*_ (*λ*_*c*_ ≥ 80 mm)	2.9	2.5	2.9	2.5	3.8	3.2
*K*(*h* = − 3cm) (mm/hr) (All)	12.4	6.7	3.0	2.6	6.5	5.0
*K*(*h* = − 3cm) (*λ*_*c*_ < 80 mm)	16.5	10.6	4.5	4.1	8.7	8.0
*K*(*h* = − 3cm) (*λ*_*c*_ ≥ 80 mm)	2.2	2.1	2.2	2.0	2.8	2.5
*K*(*h* = − 6cm) (mm/hr) (All)	5.1	3.7	2.1	1.9	3.8	3.2
*K*(*h* = − 6cm) (*λ*_*c*_ < 80 mm)	6.5	5.1	2.8	2.6	4.8	4.4
*K*(*h* = − 6cm) (*λ*_*c*_ ≥ 80 mm)	1.8	1.7	1.7	1.6	2.1	1.9

### Macropore flux, infiltration rate, and saturated hydraulic conductivity

Considering the data of the first segment, the *Q*_macro_ average values of 0.75 and 0.47 were calculated for PF and MF, respectively (Table 4). The indicated 50% difference was also observed in the third segment, where *Q*_macro_ values of 0.62 and 0.42 were recorded for PF and MF, respectively. However, an exception could be found in the results for the second segment; contrary to the general expectation, the *Q*_macro_ of MF was approximately equal to that of PF evident from Q_macro_ values of 0.65 and 0.66 representing PF and MF, respectively. Within the whole data set, the *Q*_macro_ values of 0.67 and 0.57 for PF and MF, respectively, were significantly different at *p* < 0.05 by *t*-test and *p* < 0.01 by WM-test (Table 3). The geometric means of *I*(*h* =  − 0cm) within the whole data set were 42.1 and 15.1 mm/hr for the PF and MF, respectively, while for the *K*_*s*_ they were 12.8 and 2.7 mm/hr for the PF and MF, respectively. Thus, the *I*(*h* =  − 0cm) and *K*_*s*_ were generally well associated with the inverse *λ*_*c*_, as was also attested to by the significant differences at *p* < 0.01 by a *t*-test and an MW-test of the lognormally transformed data of *I*(*h* =  − 0cm) and *K*_*s*_ (Table 3). The largest differences in *I*(*h* =  − 0cm) values were observed within segment 1, which showed geometric means of 92.6 and 12.0 mm/hr for PF and MF, respectively (Table 4). Meanwhile, the lowest difference in *I*(*h* =  − 0cm) was observed in segment 2, as indicated by the similar *I*(*h* =  − 0cm) values of 22.9 and 17.3 mm/hr for PF and 2, respectively. A similar trend was observed for the *K*_*s*_ values. The *K*_*s*_ values (segment 1/segment 2) were 47.9/7.3 and 2.9/2.9 mm/hr for the PF and MF, respectively. Table 4 also shows that the differences in infiltration rates and *K*_*s*_ values between PF and MF were reduced at tensions of −3 and −6 cm. In segment 2, the average values of *I*(*h* =  − 6cm) and *K*(*h* =  − 6cm) were similar for both PF and MF. Pearson’s correlations between the hydraulic variables (Table 5) showed a moderate negative correlation of the Q_macro of the PF with *I*(*h* =  − 6cm) and *K*(*h* =  − 6cm), and moderate positive correlation with *I*(*h* =  − 0cm). The Q_macro of the MF was only correlated *I*(*h* =  − 0cm). The infiltration rate and hydraulic conductivities values of MF and PF were intercorrelated with each other at all supply tensions. A strong positive correlation was also indicated between *α* (inverse of *λ*_*c*_) and *I*(*h* =  − 0cm) for the PF and the whole dataset.

**Table 5:** Pearson’s correlations between the hydraulic variables.

		Infiltration Rate (I)				Hydraulic conductivity		
	Q_macro	*I*(*h* = − 6cm)	*I*(*h* = − 3cm)	*I*(*h* = − 0cm)	*α* (1/cm)	*K*(*h* = − 6cm)	*K*(*h* = − 3cm)	*k*(*h* = − 0cm)
	*All data set*							
Q_macro	1.00							
*I*(*h* = − 6cm)	−0.18	1.00						
*I*(*h* = − 3cm)	−0.05	0.86	1.00					
*I*(*h* = − 0cm)	0.55	0.42	0.58	1.00				
*α* (1/cm)	0.25	0.36	0.69	0.57	1.00			
*K*(*h* = − 6cm)	−0.06	0.93	0.95	0.53	0.63	1.00		
*K*(*h* = − 3cm)	0.00	0.76	0.98	0.59	0.77	0.90	1.00	
*K*(*h* = − 0cm)	0.03	0.57	0.90	0.57	0.74	0.74	0.95	1.00
	*PF data*							
Q_macro	1.00							
*I*(*h* = − 6cm)	−0.44	1.00						
*I*(*h* = − 3cm)	−0.27	0.85	1.00					
*I*(*h* = − 0cm)	0.47	0.31	0.51	1.00				
*α* (1/cm)	0.11	0.24	0.64	0.52	1.00			
*K*(*h* = − 6cm)	−0.37	0.97	0.94	0.41	0.45	1.00		
*K*(*h* = − 3cm)	−0.21	0.76	0.99	0.54	0.72	0.88	1.00	
*K*(*h* = − 0cm)	−0.10	0.56	0.91	0.56	0.77	0.71	0.95	1.00
	*MF data*							
Q_macro	1.00							
*I*(*h* = − 6cm)	−0.06	1.00						
*I*(*h* = − 3cm)	−0.05	0.98	1.00					
*I*(*h* = − 0cm)	0.61	0.43	0.43	1.00				
*α* (1/cm)	0.02	−0.18	0.01	−0.01	1.00			
*K*(*h* = − 6cm)	0.04	0.82	0.92	0.42	0.37	1.00		
*K*(*h* = − 3cm)	0.02	0.74	0.86	0.37	0.47	0.99	1.00	
*K*(*h* = − 0cm)	0.01	0.67	0.81	0.32	0.55	0.97	0.99	1.00

### Soil Texture

Sandy loam and loamy soils are the dominant soil types in the study area (Table 1). Nevertheless, clay loam soils were found in a few locations. The average clay content values for PF for all segments were above 10% (Table 4). The soils in PF within the first segment demonstrated an average clay content of 15.8% compared to the 8% clay content of the MF. Soils in MF within the third segment were also characterized by low clay content of an average of 5.6%. However, a similar trend was not observed in the second segment, attested to by clay contents of 11.8 and 16.6 for PF and MF, respectively. In fact, the average clay content of PF and MF for the entire dataset was not statistically different (Table 3)

The average sand contents for all sites were greater than 50%. Differences between PF and MF were found in the three segments, however, similar to clay faction, there was no statistical difference between PF and MF for the entire data set.

### Organic matter

**Figure 6:**
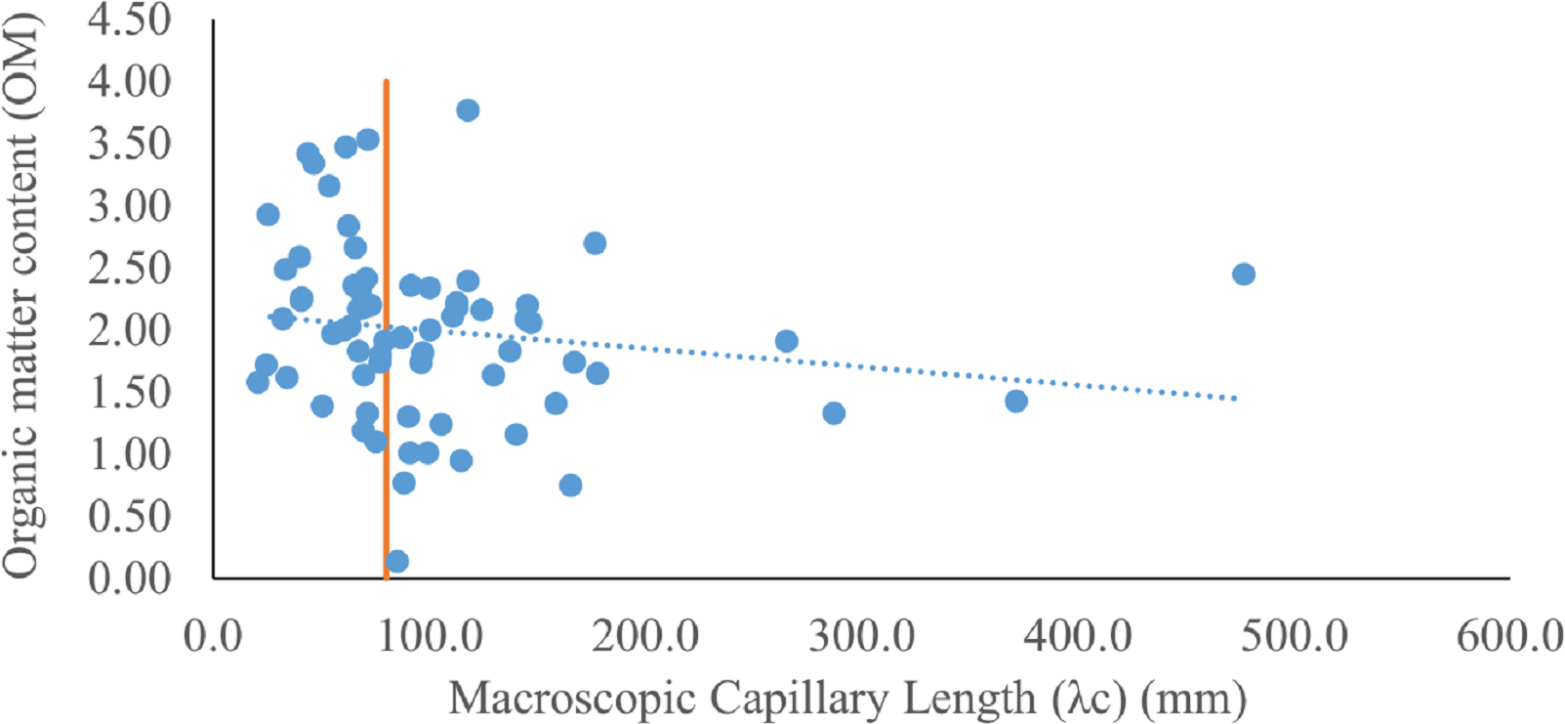
A scatter plot of organic matter content (OM) and macroscopic capillary length (*λ*_*c*_) (mm) is shown. The dashed line indicates the decreasing trend of OM with increasing *λ*_*c*_. The vertical line separates the data points of the PF (*λ*_*c*_ < 80 mm) from that of the MF (*λ*_*c*_ > 80 mm).

The results (Tables 3 and 4) showed that OM average values of PF were larger than that of MF. The OM values of the PF/ MF were 2.9/1.4, 2.3/1.9, 1.7/1.6 and 2.2/1.8 for segments 1, 2, 3 and entire study area, respectively. In addition, the student’s *t*-test and MW-test (Table 3) confirmed that the difference between the two data groups (whole data set) is statistically significant at *p* < 0.01. In general, the OM demonstrated a decreasing trend with increasing *λ*_*c*_ values (Fig. 6). Thus, indicating a negative association between OM and *λ*_*c*_.

### Soil Organic Carbon/clay fraction (SOC/clay)

**Figure 7:**
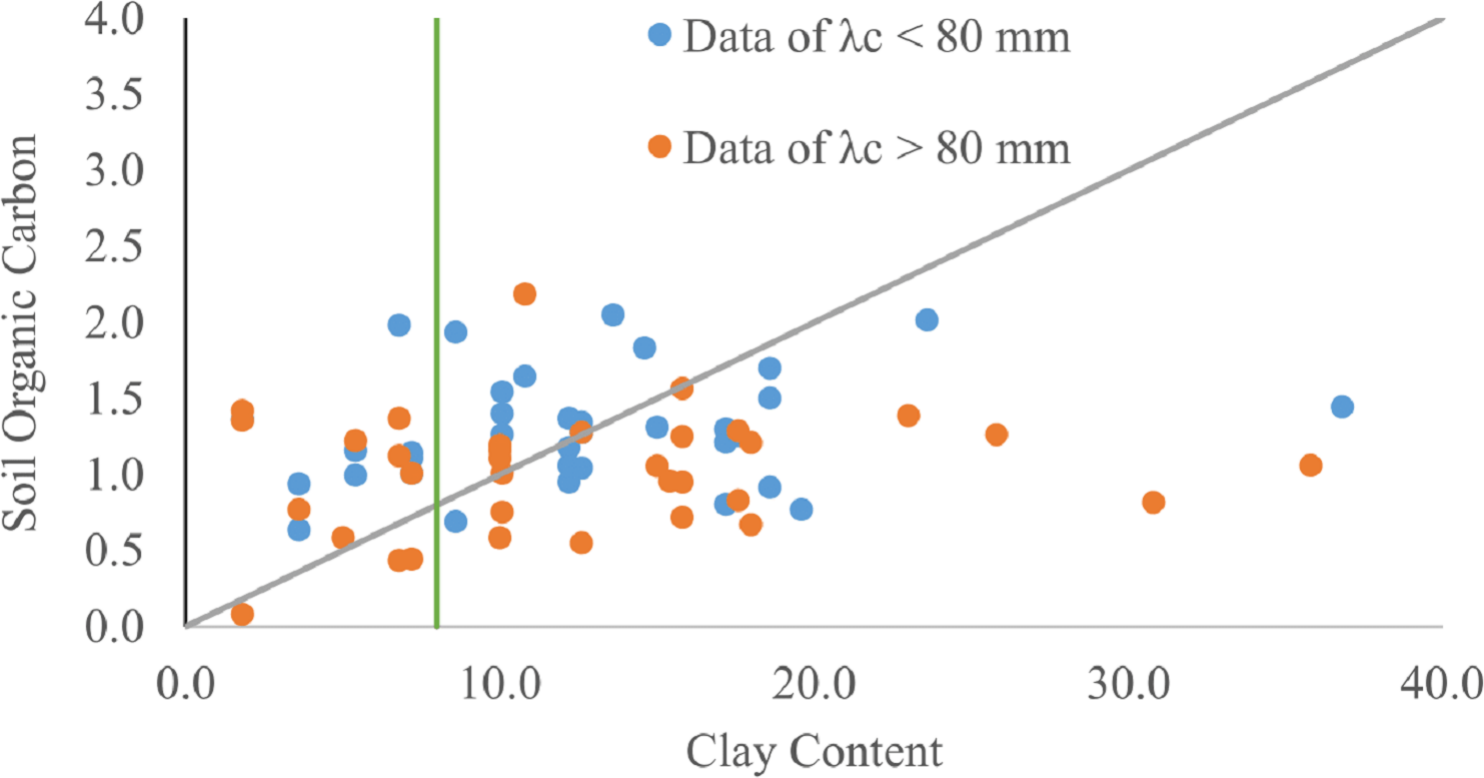
A scatter plot of the soil organic carbon and clay content is shown. The inclined line represents the 1:10 SOC/Clay ratio. The horizontal line indicates 8% clay content.

**Figure 8:**
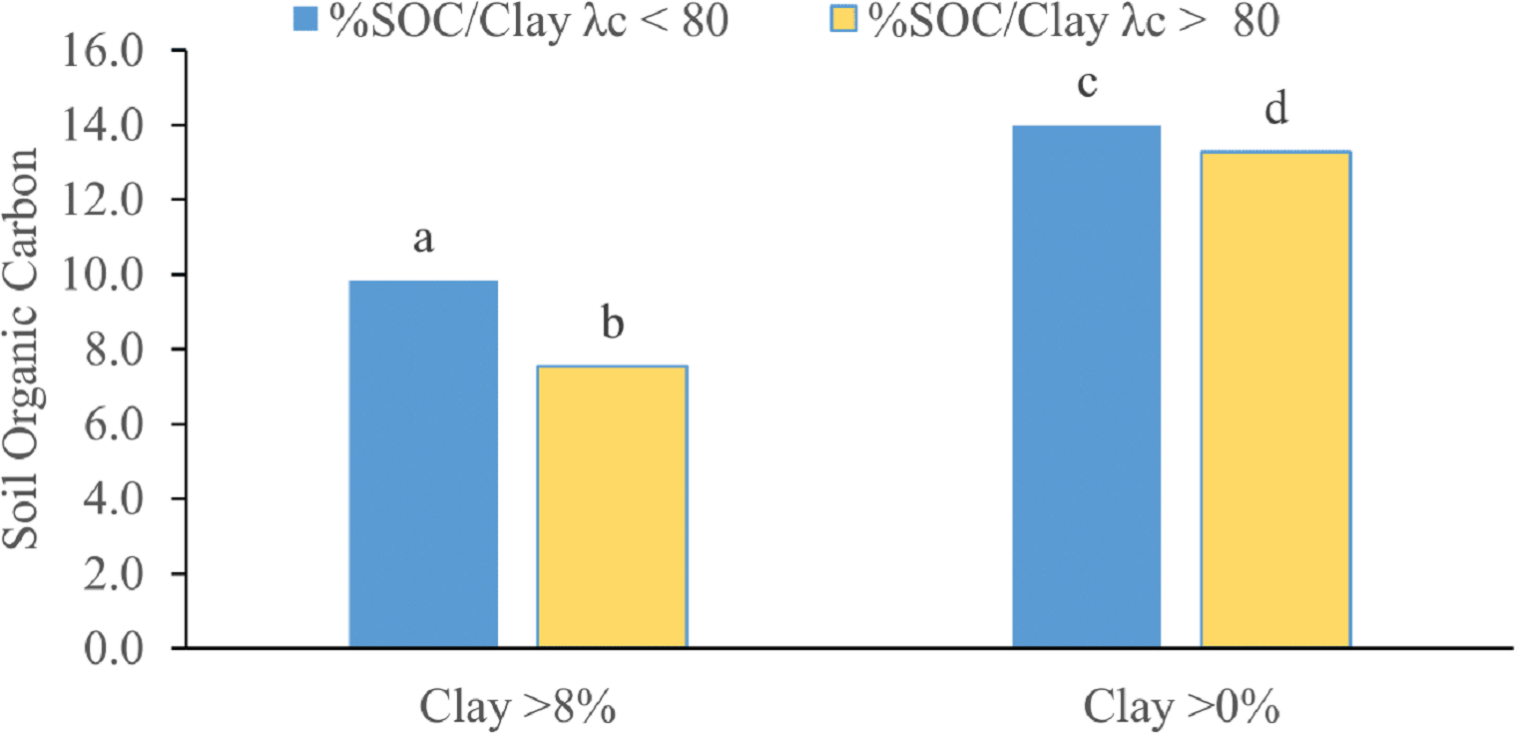
Two comparisons between the averages of SOC/Clay ratio: (1) between PF (*λ*_*c*_ < 80) and MF (*λ*_*c*_ > 80) of clay >0% for both groups. (2) between PF and MF of clay >8% for both groups. Similar letters indicate no statistical significance. Different letters indicate a significant MW-test; the null hypothesis (of no statistical difference) is rejected at *p* < 0.05.

The scatter plot of SOC versus clay content (Fig. 7) showed that the PF data are close to the 1:10 (SOC/clay) saturation line, whereas most of the MF data are below the saturation line except for soils with a clay content greater than 8%. This suggests that the SOC/clay ratios that were greater than 10% within MF were the result of low clay content. The SW and AD tests performed for SOC/clay for the PF and MF data sets returned *p* < 0.01, indicating that the normality assumption for the SOC/clay data cannot be accepted. Therefore, the MW-test was used to test the significance of the SOC/clay of the PF and MF data set. The average SOC/clay ratio of the PF (14%) was larger than that of the MF (13.3%). The MW-test produced a *p*-value of 0.047, which is close to the critical *p*-value level of 0.05 (Fig. 8). Further investigation of the SOC/clay ratio was carried out through the exclusion of soils with clay content below 8%. The averages of the newly amended SOC/clay ratio were 9.8 and 7.5% for the PF and MF, respectively. The MW-test indicated that the differences between the amended groups were statistically significant at *p* < 0.05 (*p* = 0.021).

## Discussion

### Macropore flux

Macropores are major pathways for preferential flow and transport. Thus, high *Q*_macro_ values are expected at sites that are susceptible to preferential flow. However, this is anticipated due to the empirical correlation between the flow capacities at the supply tension ranges of 0 to −3 cm and −3 to −6 cm, measured by Q_macro_ and *λ*_*c*_, respectively (e.g., Jarvis et al., 2013). The significant difference between the Q _macro_ values of PF and MF reflects the presence or absence of preferential flow as deduced from *λ*_*c*_ values (Table 3). The test of significance also indicated that high *I*(*h* =  − 0cm) values were associated with PF, which can be inferred from the considerable difference between *I*(*h* =  − 0cm) of PF and MF within segments 1 and 3. However, the influence of preferential flow on *I*(*h* =  − 0cm) was not clearly observed in segment 2, where the observed average *I*(*h* =  − 0cm) values were 27.0 and 25.8 mm/hr for PF and 2, respectively. These values are considered to be the upper end of the *I*(*h* =  − 0cm) range reported by Brouwer et al. (1988). Thus, some aspects of the preferential flow and transport may exist in soils located within the second segment, even when the preferential flow process is not fully apparent.

Also, the relatively low *K*_*s*_ values of segment 2 (as compared to those of segments 1 and 3) can facilitate preferential flow due to the build-up of saturation in the soil as a result of the slow movement of soil water, which subsequently induces macro-pore flow (Koestel et al., 2013). Furthermore, Larsbo, Koestel & Jarvis (2014) showed that macropore flow is not necessarily related to large macroporosities. Preferential flow can occur in soils of low macroporosity through the few preserved macropores. This explains the proximity of *I*(*h* =  − 0cm) for PF and MF within segment 2 despite the disparity in *λ*_*c*_ values (A.M. of 65.6 and 136.1 for PF and MF, respectively). In fact, the reduction of macroporosity increases the severity of preferential flow because the elimination of some macropores and the decrease of near-saturated hydraulic conductivity can quickly bring the soil water content to saturation, subsequently activating the remaining macropores that at saturation can conduct a larger amount of water than the soil matrix, thus dominating the flow in the soil profile. In this study, the *K*(*h* =  − 6cm) and *I*(*h* =  − 6cm) values were negatively correlated with the *Q*_macro_ values of the PF data set. Thus, high *K*(*h* =  − 6cm) can minimize or prevent the activation of soil macropores by keeping the soil water content near saturation. However, high irrigation application rates that exceed the intake rate of the soil matrix or intense rainfall events will probably lead to soil saturation and preferential macropore flow. Rahbeh, Srinivasan & Mohtar (2019) indicated that 20 mm of daily rainfall was enough to trigger preferential flow in a small irrigated site near the KTP.

### Role of organic matter and soil texture

Soil analysis of OM and soil texture are independent of the in situ tension infiltrometer measurements. Therefore, the results of the soil textual analysis and OM were examined for possible association with the preferential flow. The difference between the average clay contents of PF and MF was insignificant (Table 3). Therefore, a direct correlation between clay content and preferential flow cannot be inferred from the results. However, it is worth noting that, in this study, preferential flow was associated with clay content of between 11–16%. This is in agreement with Iversen et al. (2012) and Ghafoor et al. (2013), who reported a positive correlation between preferential transport and fine-textured soils. Also, it aligns with the threshold of 8% clay for macropore formation as suggested by Koestel, Moeys & Jarvis (2012).

However, the positive association of clay content with preferential flow was not observed in the second segment, attested to by clay content of 11.8 and 16.6 for PF and MF, respectively. Apparently, low clay content may impede preferential flow. However, high clay content does not necessarily create favorable conditions for preferential flow (Koestel, Moeys & Jarvis, 2012).

Preferential flow is also influenced by OM (Dexter et al., 2008). The role of OM as a cementing agent is well known (e.g., Hillel, 2004). OM improves the structure and physical properties of soils. It facilitates the formation of soil macropores, thus increasing the infiltrability of the soil as well as improving the aeration and redistribution of soil water. These effects are favorable from an irrigation point of view, but may also increase the potential of preferential flow, thus leaving the rechargeable groundwater aquifer susceptible to environmental risks. The positive association between OM and preferential flow is indicated by the significant difference in OM between PF and MF (Table 3), and also by the decreasing OM trend with increasing *λ*_*c*_ values (Fig. 6).

The differences between the SOC/clay ratios of soils associated with *λ*_*c*_ < 80 mm and *λ*_*c*_ ≥ 80 mm were marginally significant. Therefore, SOC/clay may not be a sufficient indicator of susceptibility to preferential flow (Fig. 8). The lack of statistical significance may be attributed to high SOC/clay ratios resulting from clay content below the 8% minimum threshold required for soil aggregation and the formation of macropores (Koestel, Moeys & Jarvis, 2012), thus negating the influence of OM. The exclusion of soils with clay content below 8% increased the statistical difference in SOC/clay ratio between PF and MF (Fig. 8). Thus, the combination of an SOC/clay ratio close to or greater than 10% and clay content above 8% may be used as a criterion for indicating susceptibility to preferential flow.

Apparently, for the types of soils along the ZR, the macropore formation favors fully complexed clay content or soils with an SOC/clay ratio slightly greater than 10%, provided that the soil has a clay content of 8%, which is the minimum required for aggregation. These soils are generally more susceptible to preferential flow than are soils of low clay content. Therefore, the observation that the preferential flow was influenced interdependently by OM and clay content aligns with the results obtained by Dexter et al. (2008), Zaffar & Lu (2015), and Larsbo et al. (2016).

It should be noted that previous studies indicated a positive correlation between preferential flow and clay content, whereas OM was either secondary in importance to clay content or essential to the stability of the soil structure but did not directly correlate with macropore formation (Karup et al., 2016). Furthermore, Ghafoor et al. (2013) and Larsbo et al. (2016) observed that high OM reduced preferential transport in soils that were rich in clay. The lack of a correlation between OM and preferential flow may also be attributed to the lack of variation in OM or SOC/clay (Koestel et al., 2013; Karup et al., 2016). However, the more important role of OM in this study, as compared to its role in previous studies, can also be attributed to climate differences (Jarvis et al., 2013). Most studies of preferential flow and transport were conducted in temperate to humid climates with a mean annual precipitation range between 300 and 1,100 mm (Jorda et al., 2015). By contrast, the ZR is an arid region characterized by an annual mean rainfall of less than 150 mm. Jorda et al. (2015) indicated that the temperature and precipitation norms are key controls of near-saturated hydraulic conductivity. Furthermore, a regional analysis using soil data compiled from across the USA (Hirmas et al., 2018) showed greater macropore formation in dry regions than in wet regions. This was indicated by the inverse relationship between macroporosity and precipitation variables that included an average magnitude of single rainfall events and mean annual precipitation. They inferred that the decline of macroporosity in humid regions may be attributed to intense rainfall events that caused soil macropores to shift into smaller pores.

Climatic conditions may also influence local levels of OM and clay content. Clay content is proportional to annual rainfall. Therefore, soils in temperate to humid regions are usually richer in clay than are soils in arid regions (Jenny, 1941). Furthermore, high clay content enhances the storage of SOC (Hartati & Sudarmadji, 2016). In this study, clay content and OM were less than 20 and 2%, respectively, in most locations. These values are generally lower than the range of values reported from temperate regions. For example, Ghafoor et al. (2013) reported a range of clay content between 5 and 40% and an OM range between 1.5 and 4%. Also, Larsbo et al. (2016) collected undisturbed soil columns from a site characterized by high clay content (64–70%) and a variable OM range of between 4 and 15%. Larsbo et al. (2016) explained that high OM induced the increased formation of smaller macropores with a diameter of 200–600 µm, which resulted in the reduction of preferential transport. Nemes, Rawls & Pachepsky (2005) showed that high clay content drives the pedotransfer functions into an inverse correlation between OM and *K*_*s*_; however, for low OM and clay content, *K*_*s*_ is positively correlated with OM. Rawls, Nemes & Pachepsky (2004) suggested that *K*_*s*_ increases with an increase of OM in clay content of less than 30%. At a higher clay content, the relation between OM and *K*_*s*_ becomes more complex. Therefore, it is plausible that at low OM and clay content (e.g., 10–20% clay content and 0.5–2% SOC), the preferential flow was positively associated with OM due to the formation of macropores when SOC/clay approached or exceeded the 1:10 saturation level.

However, macropores are also a reflection of the activity of soil fauna and plant roots (Beven & Germann, 1982). Soil OM can be an outcome rather than the cause of preferential flow. In fact, the preferential flow pathway can alter the distribution of OM (Hagedorn et al., 2015). Cheng et al. (2014) observed higher OM in the preferential flow pathways than in the soil matrix, which they attributed to the presence of decaying roots in the macropores. The ZR valley is intensely cultivated. Farmers run up to three cultivation cycles per year. The intense irrigated agriculture activity increases the lateral and vertical distribution of roots, and also enhances the growth of worms and insects. The steady biological activity in the soil subsurface generates new pathways for express water and contaminant movement and, simultaneously, the accumulation of new organic matter in the soil.

## Conclusions

The susceptibility of soil near the ZR to preferential flow was investigated using in situ tensiometer measurements combined with soil analysis of OM and soil texture. The Wooding (1968) was used to calculate *λ*_*c*_. In addition, determinations were made for *Q*_macro_ and *I*(*h* =  − 0cm). *λ*_*c*_ values of less than 80 mm, seen as an indicator of preferential flow, were observed at various locations along the ZR and were dominant in soils located near the KTP. This suggests the existence of preferential flow along the ZR and raises further environmental concerns related to the leaching of solute and contaminants, as *Q*_macro_ and *I*(*h* =  − 0cm) were significantly correlated with preferential flow.

The study showed that preferential flow may be characterized by the combination of clay and OM. The influence of clay content on the potential of preferential flow was apparent, but was not significantly associated with *λ*_*c*_ values. However, OM was positively correlated with preferential flow, which suggests that OM may limit the aggregation and formation of macropores. The consideration of the SOC/clay ratio showed that preferential flow favored soils with fully complexed clay content, especially soils with a clay content greater than 8% and an SOC/clay ratio above 10%. These findings confirm the inter-correlation of soil OM with preferential flow and suggest that the SOC/clay of 1:10 is a useful criterion for the initial screening of the potential of preferential flow in soils adjacent to the ZR.

##  Supplemental Information

10.7717/peerj.8057/supp-1 Data S1 Raw data Note: the coordinates of the location were redacted to protect the privacy of the farmers.

## Additional Information and Declarations

### Field Study Permissions

The following information was supplied relating to field study approvals (i.e., approving body and any reference numbers): Soil samples were collected with authorization from the farm owners. Permission to collect samples from lands owned by Agro Aqua Industries was obtained from Issa A. Wir. Permission to sample various farms came from Raed Al-Qassem, Riyad Alimat, Salman Alimat, “Abu Abrahim” Al-Mashaqbeh, Subhi Hassan, Ali Alzyoud, Mohammad Alzyoud, Samer Alzyoud, Mohammad Masoud, Abrahim Nayef, and Hamza Al Khawaldah. Other farmers were present when the measurements were conducted, and we obtained authorization directly from them at the time of sampling. No site was accessed unless the landowner was present or provided his/her consent over the phone.

### Data Availability

The following information was supplied regarding data availability: The raw data is available as a Supplemental File.
